# Case Report: Smoking as the risk factor of persistent STEMI after primary percutaneous coronary intervention: how it could be happen?

**DOI:** 10.12688/f1000research.109757.2

**Published:** 2023-07-25

**Authors:** Yusra Pintaningrum, Ricky Setiadi Yusuf, Baiq Hanida Aolia Ramdani, Shadiqa Rana Putri, Dwi Astuti Wulandari

**Affiliations:** 1 Cardiovascular Intervention, West Nusa Tenggara General Hospital, Mataram, West Nusa Tenggara, 84371, Indonesia; 2 Cardiovascular, Mataram University, Mataram, West Nusa Tenggara, 83126, Indonesia

**Keywords:** Smoking; no-reflow phenomenon; STEMI

## Abstract

Background

Acute coronary syndrome (ACS) is a common disease. Smoking may increase the risk of ACS. The most advantageous therapy is percutaneous coronary intervention. This therapy may fail which is no-reflow phenomenon as the result.

Total occlusion may increase the risk of no-reflow phenomenon which it could be worse with smoking as the habits. ST-elevation myocardial infarction (STEMI) may show in electrocardiogram (ECG).

Case description

A 37-year-old male came to the hospital with chest pain as the main complaint. ECG examination showed that there was wide anterior STEMI. Coronary angiography was then done and confirmed that there was total occlusion in left anterior descending artery. After two days hospitalization, the patient developed to cardiogenic shock and lead to acute decompensated heart failure. An ECG showed there was STEMI anterior after primary PCI.

Discussion

Many chemicals agent contain in cigarette smoking and it may induce the lipid oxidation which leads to plaque deposits. Plaque that deposits in coronary artery may rupture and make thrombus occlusion. This occlusion may partial or total, when there is total occlusion, STEMI was the result. Then, releasing the occlusion is needed for this situation ant PCI may be chosen as the therapy. Patient with wide ischemia may result the no-reflow phenomenon which may lead to heart failure and shock cardiogenic as the complication.

Conclusion

Smoking may induce ACS which leads to STEMI and may increase the failure of PCI therapy. No-reflow phenomenon is the evidence of miscarriage in therapy which it may increase because of smoking.

## Introduction

Since the early 1960s, the association between smoking and cardiovascular disease (CVD) has been explored in the Framingham Heart and the Seven Countries studies. ^1^ About 26% of ACS patients were active in smoking at hospital administration until one year forward follow-up. ^1^ Acute coronary syndrome (ACS) is one of many CVDs that commonly happens. ^2^ Patients with ACS may develop total occlusion in coronary artery, so ST-elevation myocardial infarction (STEMI) as a result in ECG (Electrocradiogram). ^3^ Percutaneous coronary intervention (PCI) is considered the most promising and rewarding reperfusion strategy. ^2^ The failure in restoring myocardial reperfusion is mostly because of the no-reflow phenomenon. ^2^ The incidence of this phenomenon is about 10–54% of procedures. ^2^ This case report will discuss the correlation between smoking and the no-reflow phenomenon and how it can occur.

## Case report

**Figure 1.**
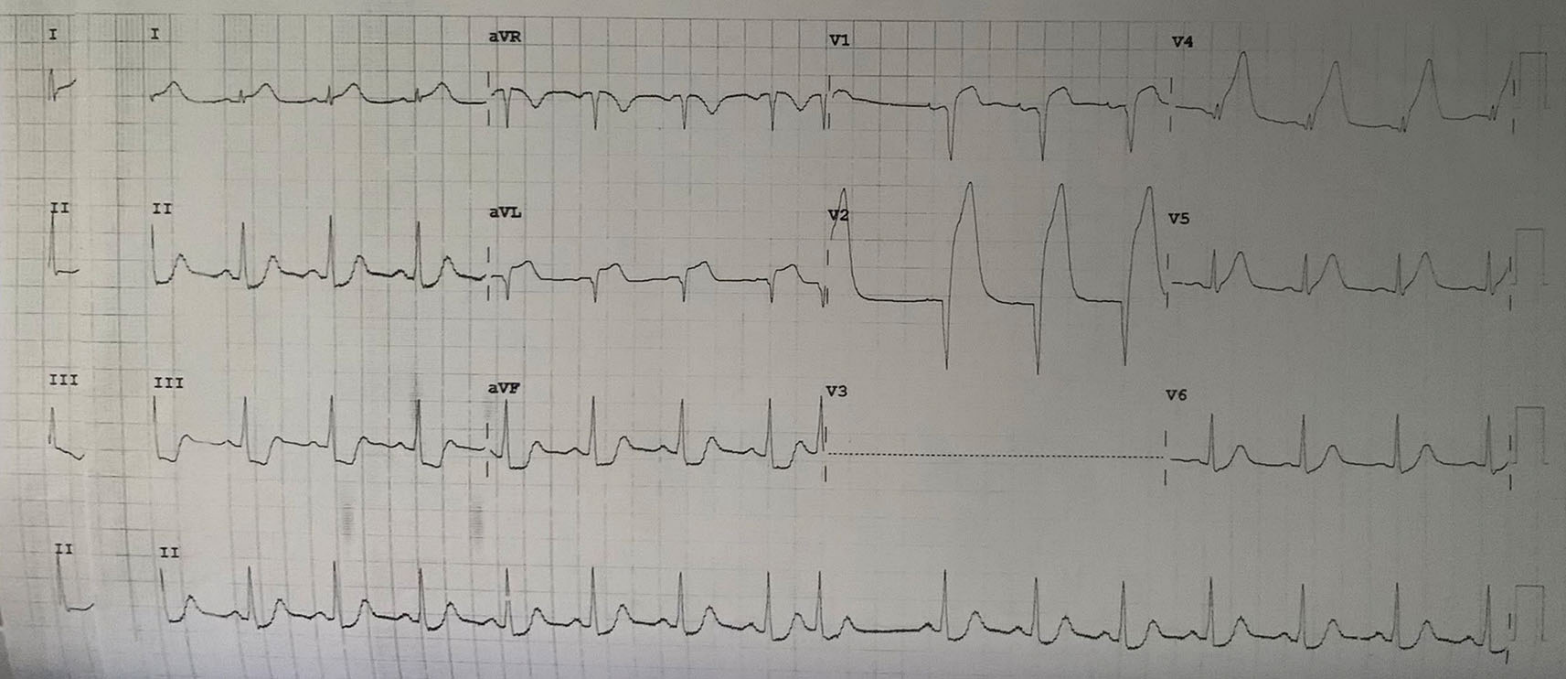
The first ECG when the patient came to the hospital.

**Figure 2.**
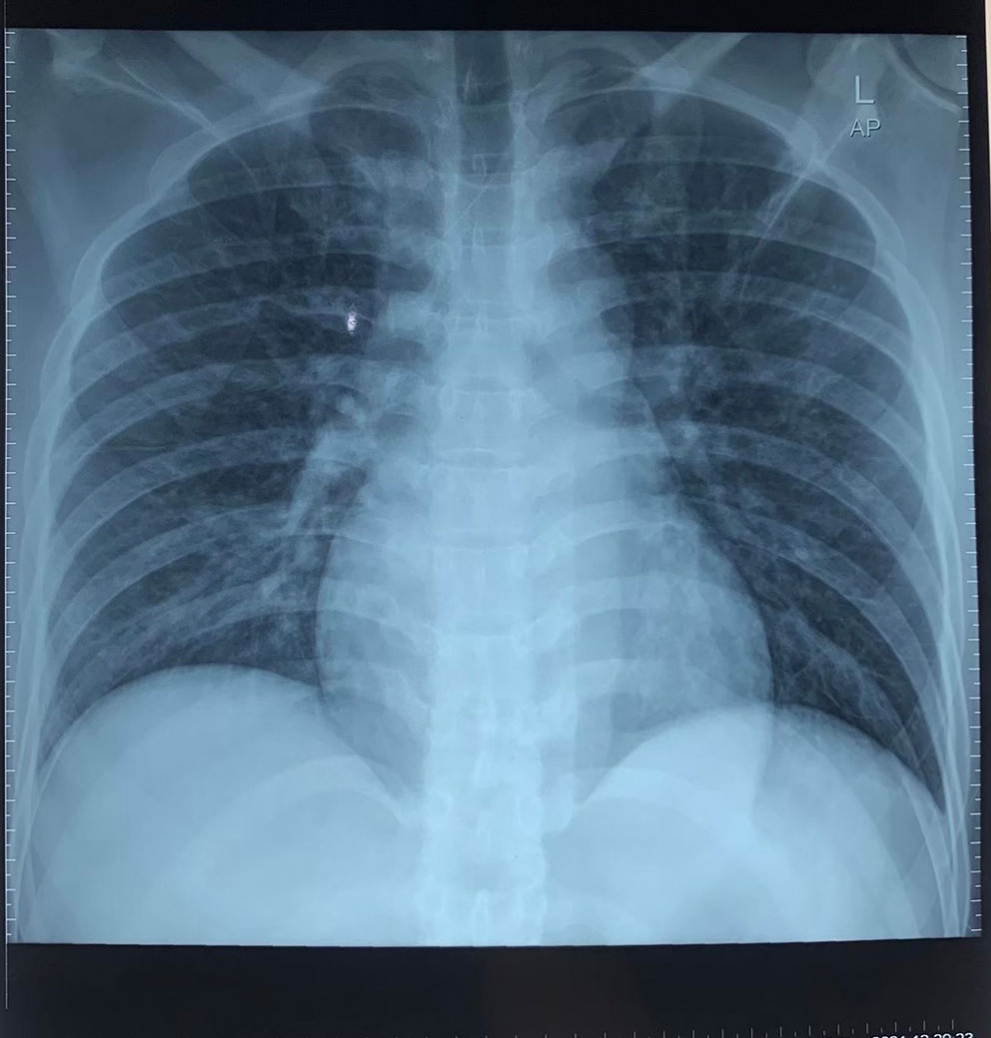
Chest radiography performed in the first hospitalization.

A 37-year-old male came to the hospital with severe chest pain as the main complaint. He is a high school teacher with no same medical or familial, or psychosocial history before. The patient said chest pain had occurred for about 3 hours before going to the hospital. He said that the pain was only in the middle of the chest, and was not reduced when resting. There are no other complaints besides chest pain. The patient said that he was a heavy smoker. In the hospital, we measured his vital signs: his temperature was 36.8°C, heart rate was 69 beats/minute, respiration rate was 23 breaths/minute, and blood pressure was 65/30 mmHg. The first electrocardiogram (ECG) was done in the hospital ( Figure 1), and there was a wide anterior acute myocardial infarction (AMI). We gave oxygenation, Ringer Lactate infusion 250 mL, dopamine 4.2 mL/hour morphine 1 mg/hours, aspirin 400 mg, clopidogrel 300 mg, and omeprazole one ampule as the first treatment for the patient. After several hours, his blood pressure increased to 130/80 mmHg. Then, the patient was transferred to perform coronary angiography, which showed total occlusion in the proximal left anterior descending artery (LAD). Also, we inserted a stent to reflow vascularization of the heart. Chest radiography was also performed, and it did not show any abnormality ( Figure 2). Blood examination was measured, and there was no abnormality.

**Figure 3.**
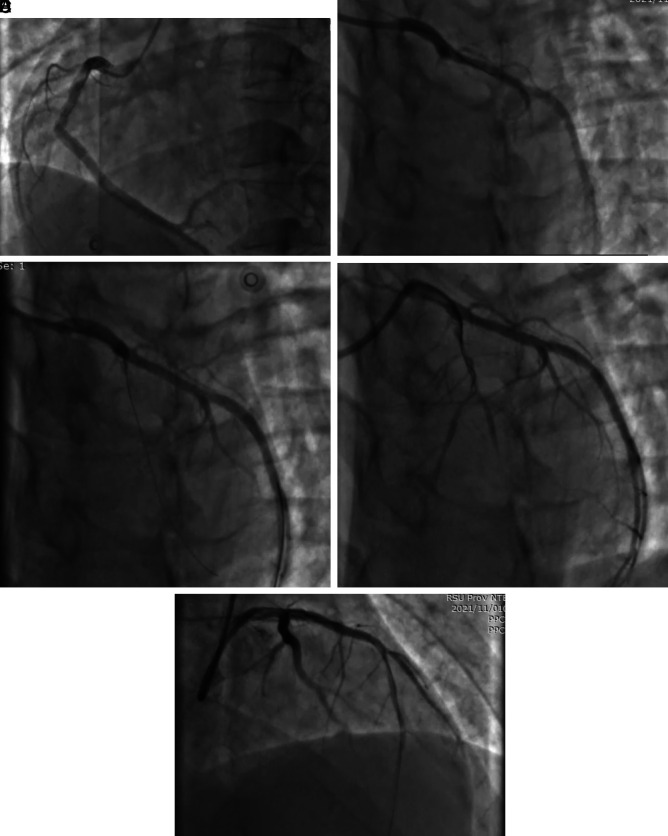
Coronary angiography. A) Normal Right Coronary Artery (RCA); B) Total occlusion in the proximal part of Left Anterior Descending Artery (LAD) and normal Left Circumflex Artery (LCX); C) Guidewire to LAD, there was flow to distal LAD (TIMI 1); D) Suddenly no-reflow and we aspirated thrombus; E) Implanting DES 3.25 × 30 mm to proximal LAD, TIMI 3 flow.

**Figure 4.**
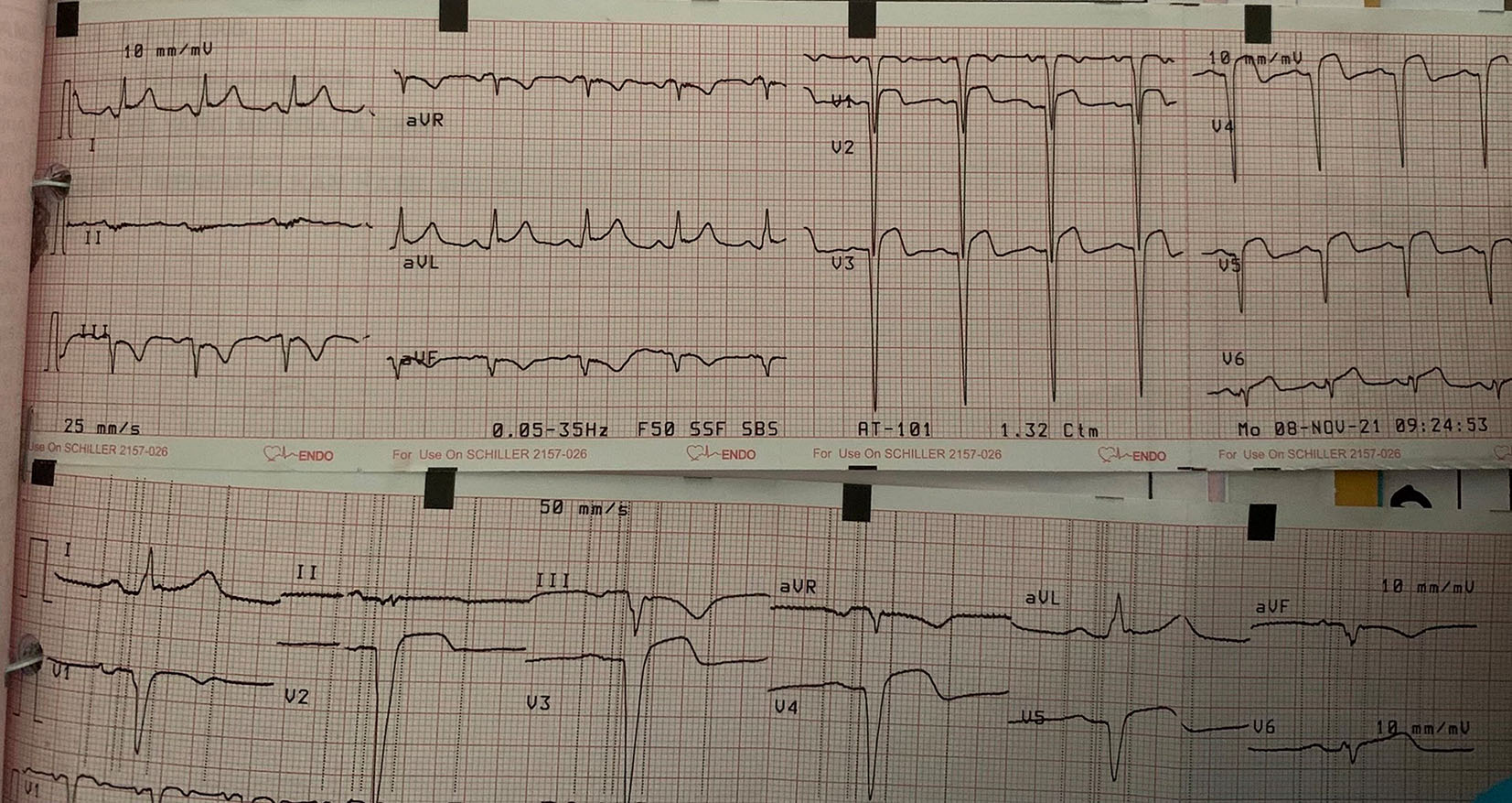
Patient's ECG when fell into ADHF and cardiogenic shock.

**Figure 5.**
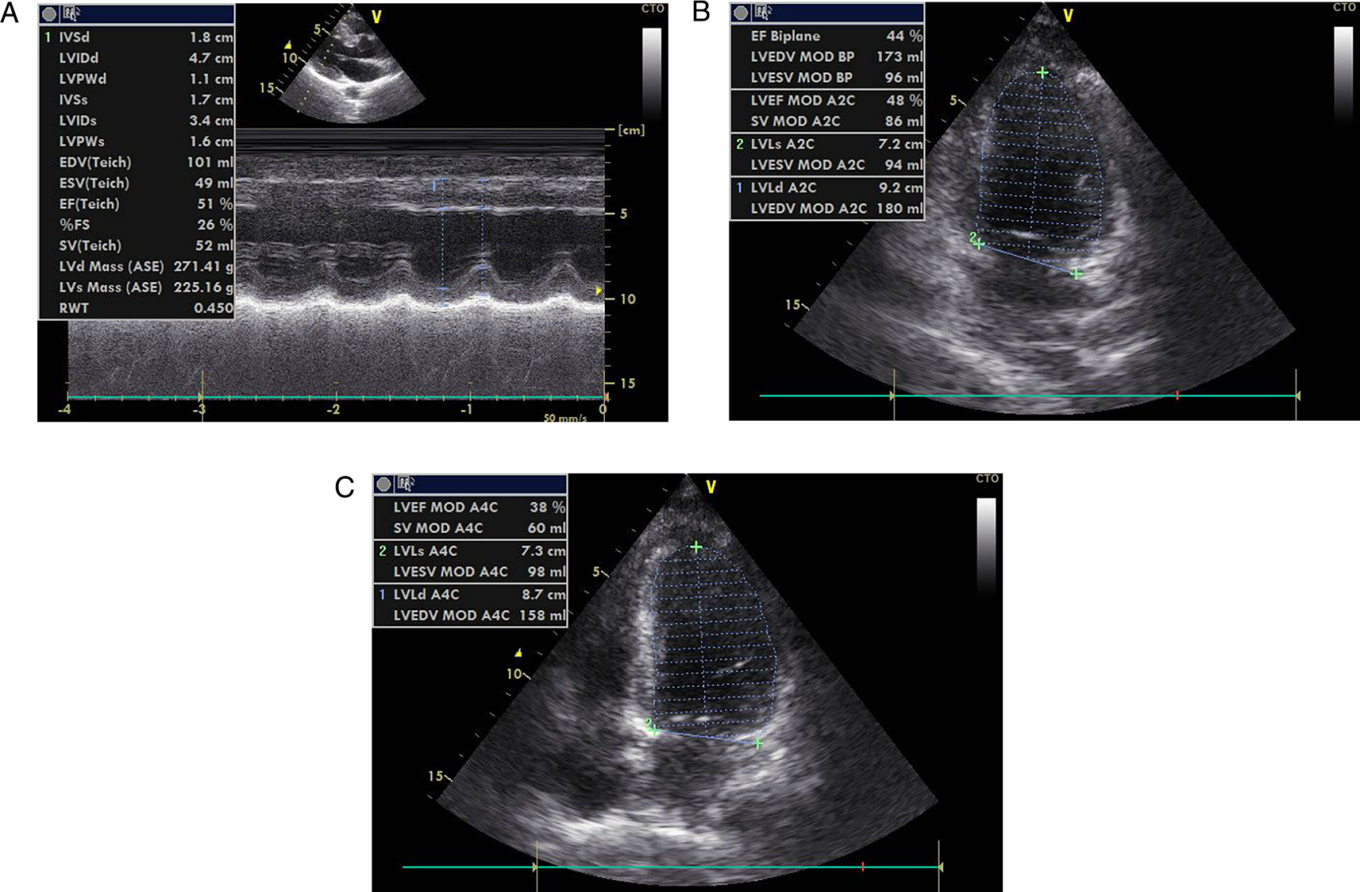
Patient's echocardiography after primary PCI.

Percutaneous coronary intervention (PCI) was conducted ( Figure 3) and after two days the patient complained of chest pain again, the same as when he came to the hospital. Then, we performed ECG, which showed wide anterior AMI ( Figure 4). After several hours the patient was unstable and fell into acute decompensated heart failure (ADHF) with cardiogenic shock. As the therapy, we combined dopamine and dobutamine five μg/kg/minute each to increase the patient’s blood pressure. Other supporting therapy was bisoprolol 2.5 mg/day, ramipril 2.5 mg/day, furosemide 20 mg/day, spironolactone 25 mg/day, atorvastatin 40 mg/day, aspirin 80 mg/day, nitroglycerin 20 μg/minute, and we changed clopidogrel to ticagrelor 90 mg/12 hours. Other supporting therapies were infusion with normal saline 500 mL/day, alprazolam 0.5 mg/day, Ivabradine 5 mg/12 hours, and ondansetron 4 mg/day. Thus, we also restricted water intake to between 1000–1200 cc/day. The echocardiography of the patient showed coronary artery disease (CAD) with anteroseptal LV hypokinetic, normal LV and mild reduce ejection fraction (EF by teach 51%) ( Figure 5). After two days, the patient was in better condition, and the next day the patient was discharged from the hospital.

## Discussion

Cigarette smoke contains about 4,000 different chemical agents considered the most complex and the least understood among cardiovascular risk factors of cardiovascular disease. ^3^ Smoking-related cardiovascular dysfunction caused by toxic components in cigarette smoke has multiple mechanisms, including increased inflammation, oxidation of low-density lipoprotein cholesterol, platelet aggregability, thromboxane production, plasma viscosity, and fibrinogen levels. Smoking also causes alteration of the function of endothelial cells and reduces oxygen supply. ^4^^,^^5^

As well as directly damaging coronary arteries, smoking also raises levels of harmful oxidized low-density lipoprotein and reduces beneficial high-density lipoprotein, thereby contributing to an increase in fatty deposits (plaque) at the site of the injury in the arteries. Smokers have higher extracellular lipid content in their plaque, which renders the plaque vulnerable to rupture. Endothelial injury and dysfunction promote platelet adhesion and lead to the formation of a blood clot: a process known as thrombosis. Tobacco smoking also induces a hypercoagulable state, increasing the risk of acute thrombosis. Smoking-mediated thrombosis appears to be a significant factor in the pathogenesis of critical cardiovascular events. ^4^

The pathogenesis and risk factors of no-reflow are still incompletely understood. Several risk factors have been associated with the risk of a no-reflow phenomenon. Smoking is one of the risk factors for the incidence of the no-reflow phenomenon. ^6^^,^^7^ However, several recent studies have shown that smokers have less reflow phenomenon after PCI. Data from Shemirani *et al*., showed that the incidence of the no-reflow phenomenon was not significantly different between smokers and non-smoker. ^2^ This finding is still controversial. Theoretically, smoking is associated with the risk of CAD and endothelial dysfunction. Nevertheless, it has been described that the causes of no-reflow are multifactorial. Therefore, smoking cannot be judged as the only factor influencing no-reflow. ^6^

No-reflow phenomenon (NRP) is the hypoperfusion of myocardial tissue after an occlusion is removed even though the epicardial coronary arteries are open and patent. NRP is quite common in patients who experience acute ST-elevation myocardial infarction (STEMI) and then receive primary percutaneous coronary intervention (PPCI). ^8^^–^^10^ The process of how NRP happens has not been clearly described. It is said that many factors influence it, such as smoking, as in our case. In addition, the presence of leukocyte infiltration, vasoconstriction, activation of inflammatory pathways, and cellular edema is associated with the phenomenon of the occurrence of NRP. ^9^^,^^10^

Based on a study by Pantea *et al*., one-third of patients with NRP experience various complications. ^8^^,^^9^ The presence of anterior STEMI and lesions in the left anterior descending artery (LAD) is associated with a high incidence of complications in these patients. ^8^ Complications include hemodynamics disturbance, cardiogenic shock, myocardial rupture, pulmonary edema, heart failure, arrhythmias, and others. ^8^^,^^9^ In this case, the patients develop cardiogenic shock and acute decompensated heart failure (ADHF). Cardiogenic shock results from long-term myocardial necrosis and secondary rupture of the free myocardial wall or interventricular septum. ^8^ In addition, in NRP, heart rhythm disturbances occur, which cause ischemia in the long term, resulting in extensive necrosis of the heart myocardium. Furthermore, changes in myocardial function can occur, where ejection fraction decreases due to modification of left ventricle (LV) function. ^8^^,^^9^ This will reduce contractility function, increasing the risk of acute pulmonary edema and acute heart failure.

## Conclusion

Smoking is one of the most common habits in people all over the world. Many adverse effects may occur because of smoking. One of them is ACS which STEMI is one of the diseases included. One of many therapies that may be done to reflow the blood’s obstruction. Percutaneous coronary intervention (PCI) is one of them, and now it is claimed as the most advantageous to reperfuse the coronary artery. Failure of this therapy may happen, and the no-reflow phenomenon is the most common. Smoking may induce the no-reflow phenomenon and may lead to heart failure, increasing the risk of cardiogenic shock.

## Data availability

All data underlying the results are available as part of the article and no additional source data are required.

## Consent

Oral informed consent for publication of their clinical details and/or clinical images was obtained from the patient. Oral rather than written consent was obtained because of the patient’s condition and education level of the family, and was approved by the ethical review board of West Nusa Tenggara Province hospital.
