# Case Report: Smoking as the risk factor of persistent STEMI after primary percutaneous coronary intervention: how it could be happen?

**DOI:** 10.12688/f1000research.109757.3

**Published:** 2024-10-29

**Authors:** Yusra Pintaningrum, Ricky Setiadi Yusuf, Baiq Hanida Aolia Ramdani, Shadiqa Rana Putri, Dwi Astuti Wulandari

**Affiliations:** 1 Cardiovascular Intervention, West Nusa Tenggara General Hospital, Mataram, West Nusa Tenggara, 84371, Indonesia; 2 Cardiovascular, Mataram University, Mataram, West Nusa Tenggara, 83126, Indonesia

**Keywords:** Smoking; no-reflow phenomenon; STEMI

## Abstract

**Background:**

Acute coronary syndrome (ACS) remains one of the leading causes of death worldwide. Smoking may also increase the risk of developing ACS. The most advantageous therapy is percutaneous coronary intervention. However, this therapy may fail because of the no-reflow phenomenon. This case report describes a young male patient admitted to the emergency department due to ST-segment elevation of myocardial infarction (STEMI), with smoking as the only risk factor.

**Case description:**

A 37-year-old male presented to our hospital with a typical chest pain. He was a heavy smoker. Electrocardiography (ECG) revealed extensive anterior STEMI. Coronary angiography revealed total occlusion of the proximal left anterior descending artery (LAD) with a high-burden thrombus. The no-reflow phenomenon occurs during Percutaneous Coronary intervention (PCI). After two days of hospitalization, the patient developed cardiogenic shock and acute decompensated heart failure. The patient was administered ticagrelor, acetylsalicylic acid, enoxaparin for three days, high-dose statins, and optimized heart failure treatment. The patient was discharged on the 7th day after admission.

**Discussion:**

Cigarette smoke chemicals may induce atherosclerosis and thickened blood in the arteries. Lipid oxidation leads to plaque formation. If plaque ruptures, it will cause thrombus occlusion. A high-burden thrombus can induce a no-reflow phenomenon, leading to heart failure and cardiogenic shock.

**Conclusion:**

Smoking may induce STEMI and tends to result in a high-burden thrombus. The no-reflow phenomenon is an evidence of miscarriage during PCI, which may increase because of smoking.

## Introduction

Since the early 1960s, the association between smoking and cardiovascular disease (CVD) has been explored in the Framingham Heart and the Seven Countries studies. ^1^ About 26% of ACS patients were active in smoking at hospital administration until one year forward follow-up. ^1^ Acute coronary syndrome (ACS) is one of many CVDs that commonly happens. ^2^ Patients with ACS may develop total occlusion in coronary artery, so ST-elevation myocardial infarction (STEMI) as a result in ECG (Electrocradiogram). ^3^ Percutaneous coronary intervention (PCI) is considered the most promising and rewarding reperfusion strategy. ^2^ The failure in restoring myocardial reperfusion is mostly because of the no-reflow phenomenon. ^2^ The incidence of this phenomenon is about 10–54% of procedures. ^2^ This case report will discuss the correlation between smoking and the no-reflow phenomenon and how it can occur.

## Case report

**Figure 1.**
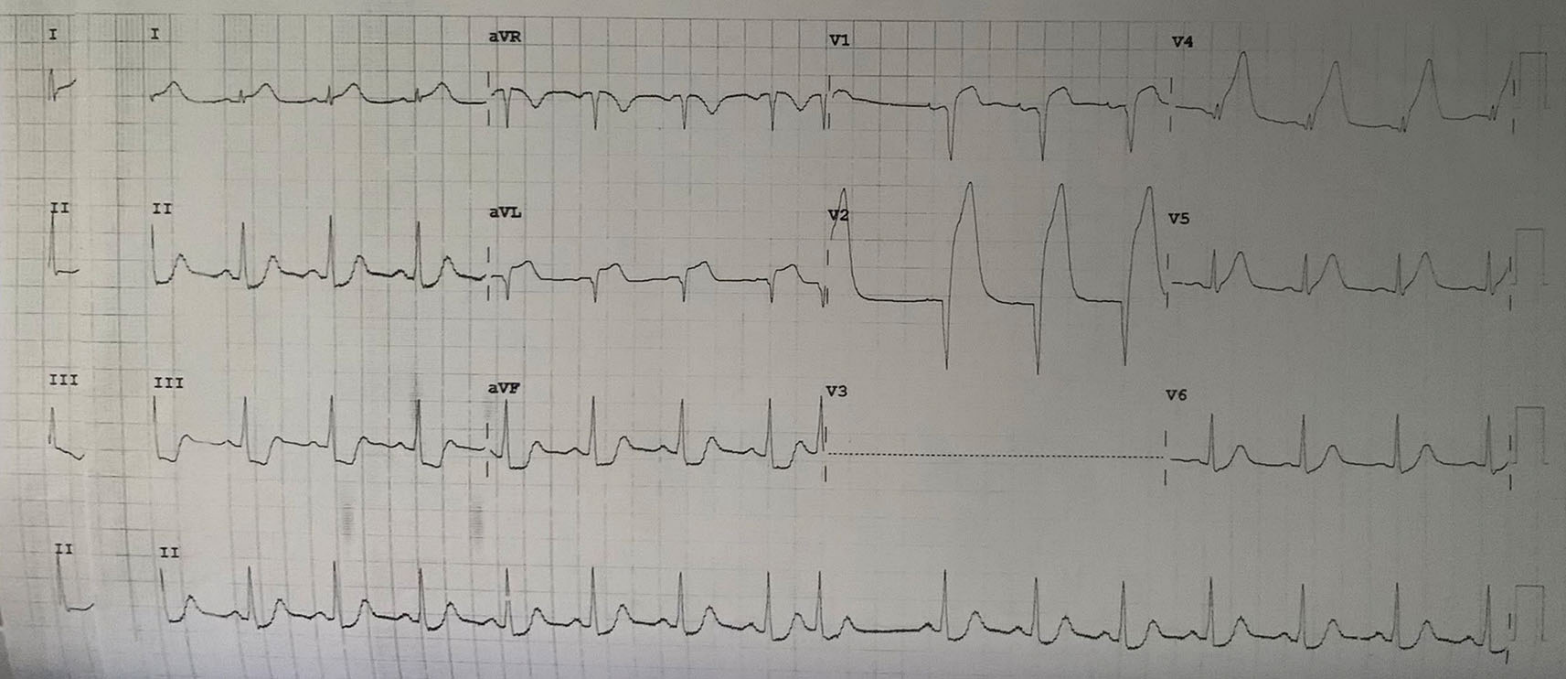
The first ECG when the patient came to the hospital.

**Figure 2.**
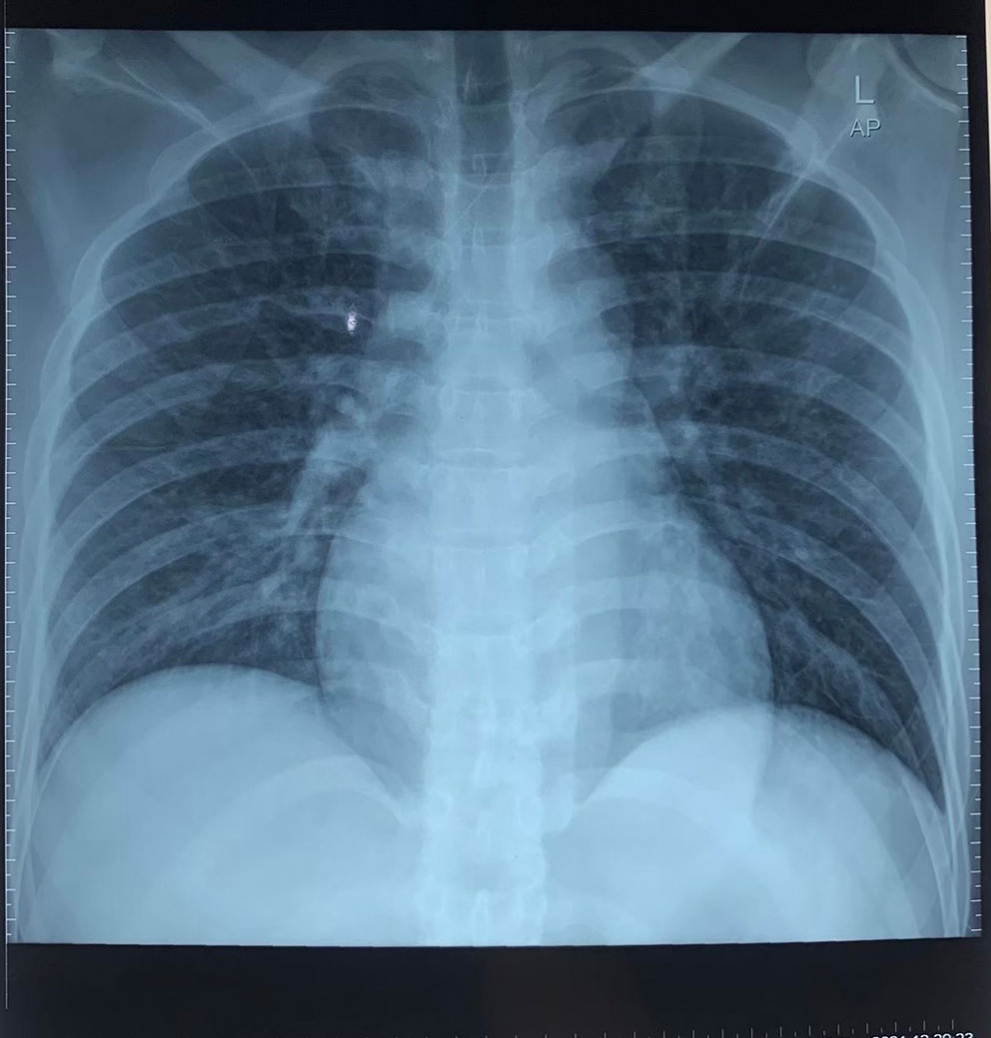
Chest radiography performed in the first hospitalization.

**Figure 3.**
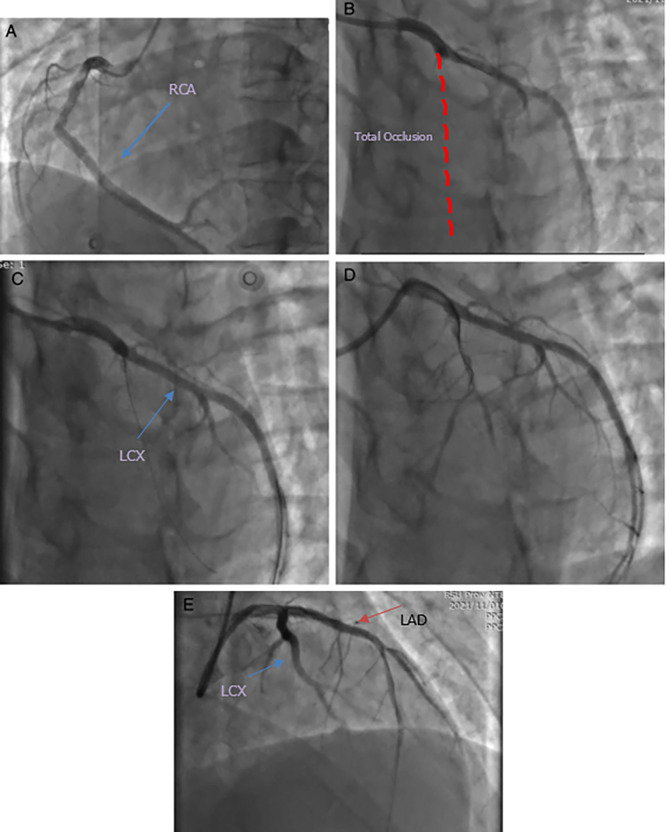
Coronary angiography. A) Normal Right Coronary Artery (RCA); B) Total occlusion in the proximal part of Left Anterior Descending Artery (LAD) and normal Left Circumflex Artery (LCX); C) Guidewire to LAD, there was flow to distal LAD (TIMI 1); D) Suddenly no-reflow and we aspirated thrombus; E) Implanting DES 3.25 × 30 mm to proximal LAD, TIMI 3 flow.

A 37-year-old male presented at our hospital with severe chest pain in the middle of the chest three hours before going to the hospital. The pain was not relieved with rest. He was a high school teacher without medical, familial, or psychosocial history. He was a heavy smoker. The patient's vital signs were as follows: temperature was 36.8°C, heart rate was 69 beats/minute, respiration rate was 23 breaths/minute, and blood pressure was 65/30 mmHg. The first electrocardiogram (ECG) was done in the hospital ( Figure 1) revealed extensive anterior acute myocardial infarction (AMI). We administered oxygenation, Ringer Lactate infusion 250 mL, dopamine 4.2 mL/hour, morphine 1 mg/hours, aspirin 400 mg, and clopidogrel 300 mg as the first treatment for the patient. After several hours, his blood pressure increased to 130/80 mmHg. Chest radiography revealed no abnormalities ( Figure 2). Blood examination revealed no abnormalities, and the patient was transferred for coronary angiography, which showed total occlusion with a high-burden thrombus in the proximal left anterior descending artery (LAD). Percutaneous coronary intervention (PCI) was performed ( Figure 3). A drug-eluting stent (DES) was implanted in the LAD; however, the thrombus returned. The thrombus was aspirated until TIMI 3 flow was achieved.

**Figure 4.**
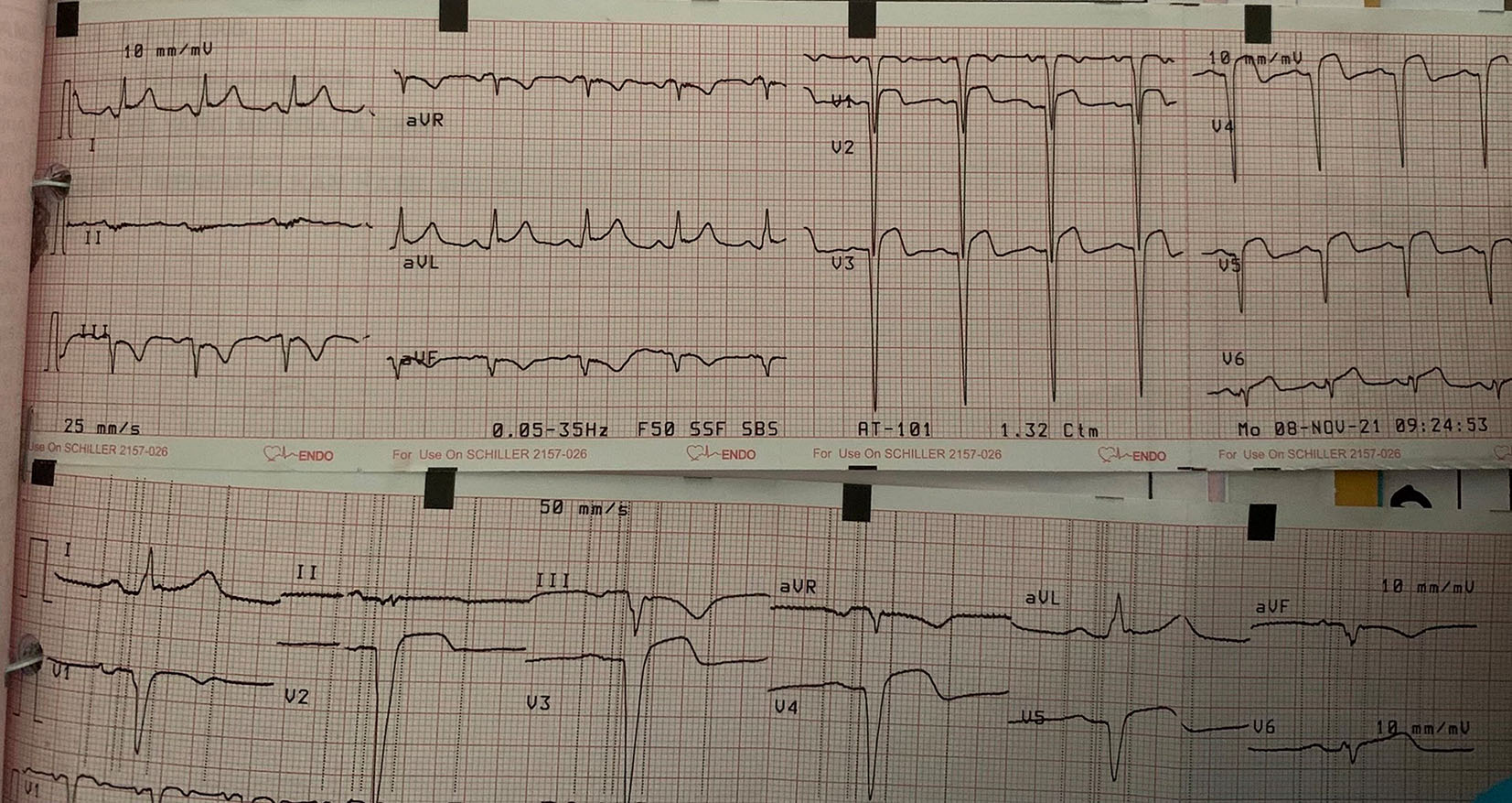
Patient's ECG when fell into ADHF and cardiogenic shock.

**Figure 5.**
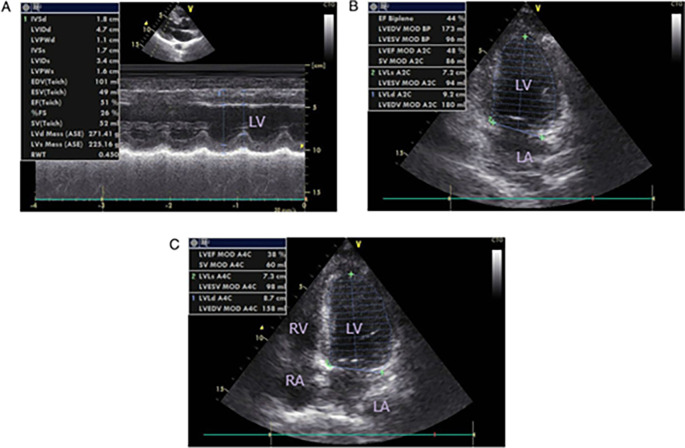
Patient's echocardiography after primary PCI.

After two days, the patient complained of typical chest pain. We then performed ECG, which showed extensive anterior STEMI ( Figure 4). After several hours, the patient became unstable and experienced acute decompensated heart failure (ADHF) with cardiogenic shock. We combined 5 μg/kg/minute dopamine and 5 μg/kg/minute dobutamine to increase the blood pressure. After the hemodynamic was stable, we started to give some medicine from low dose such as bisoprolol 2.5 mg/day, ramipril 2.5 mg/day, furosemide 20 mg/day, spironolactone 25 mg/day, atorvastatin 40 mg/day, aspirin 80 mg/day, nitroglycerin 10 μg/minute, and we changed clopidogrel to ticagrelor 90 mg/12 hours. Other supporting therapies included infusion with normal saline (500 mL/day, alprazolam 0.5 mg/day, and ondansetron 4 mg/day, if needed. The echocardiography of the patient showed coronary artery disease (CAD) with anteroseptal LV hypokinetic, normal LV and mild reduce ejection fraction (EF by teach 51%) ( Figure 5). After two days, the patient was in better condition, and the next day the patient was discharged from the hospital.

## Discussion

Cigarette smoke contains about 4,000 different chemical agents considered the most complex and the least understood among cardiovascular risk factors of cardiovascular disease. ^3^ Smoking-related cardiovascular dysfunction caused by toxic components in cigarette smoke has multiple mechanisms, including increased inflammation, oxidation of low-density lipoprotein cholesterol, platelet aggregability, thromboxane production, plasma viscosity, and fibrinogen levels. Smoking also causes alteration of the function of endothelial cells and reduces oxygen supply. ^4^^,^^5^

In addition to directly damaging the coronary arteries, smoking also increases the levels of harmful oxidized low-density lipoproteins. It reduces beneficial high-density lipoprotein, thereby contributing to an increase in fatty deposits (plaques) at the site of arterial injury. Smokers have a higher extracellular lipid content in their plaque, which renders it vulnerable to rupture. Endothelial injury and dysfunction promote platelet adhesion and lead to the formation of a blood clots, a process known as thrombosis. Tobacco smoking also induces a hypercoagulable state, which increases the risk of acute thrombosis. Smoking-mediated thrombosis appears to be a significant factor in the pathogenesis of critical cardiovascular events. ^4^

The pathogenesis and risk factors for no-reflow are not entirely understood. Several risk factors have been associated with the risk of a no-reflow phenomenon. Smoking is a risk factor for the incidence of high thrombus burden, which induces the no-reflow phenomenon. ^6^^,^^7^ However, several recent studies have shown that smokers experience less of a reflow phenomenon after PCI. Data from Shemirani *et al*., showed that the incidence of the no-reflow phenomenon was not significantly different between smokers and nonsmokers. ^2^ This finding is still controversial. Theoretically, smoking is associated with the risk of CAD and endothelial dysfunction. Nevertheless, it has been described that the causes of no-reflow are multifactorial. Therefore, smoking cannot be judged as the only factor influencing no-reflow. ^6^

The no-reflow phenomenon (NRP) is the hypoperfusion of myocardial tissue after an occlusion is removed, even though the epicardial coronary arteries are open and patent. NRP is quite common in patients who experience acute ST-elevation myocardial infarction (STEMI) and then receive primary percutaneous coronary intervention (PPCI). ^8^^–^^10^ The process by which NRP occurs has not been clearly described. As in the present case, many factors influence it, including smoking. In addition, the presence of leukocyte infiltration, vasoconstriction, activation of inflammatory pathways, and cellular edema is associated with the phenomenon of the occurrence of NRP. ^9^^,^^10^

According to a study by Pantea *et al*., one-third of patients with NRP experience various complications. ^8^^,^^9^ The presence of anterior STEMI and lesions in the left anterior descending artery (LAD) is associated with high incidence of complications in these patients. ^8^ Complications include hemodynamics disturbance, cardiogenic shock, myocardial rupture, pulmonary edema, heart failure, and arrhythmias. ^8^^,^^9^ In this case, the patients develop cardiogenic shock and acute decompensated heart failure (ADHF). Cardiogenic shock results from long-term myocardial necrosis and secondary rupture of the free myocardial wall or interventricular septum. ^8^ In addition, in NRP, heart rhythm disturbances occur, which cause ischemia in the long term, resulting in extensive necrosis of the heart myocardium. Furthermore, changes in myocardial function can occur when ejection fraction decreases due to modification of left ventricle (LV) function. ^8^^,^^9^ This reduces contractility and increases the risk of acute pulmonary edema and acute heart failure.

## Conclusion

Smoking is one of the most common habits in people all over the world. Many adverse effects may occur owing to smoking. One of these is ACS, in which STEMI is one of the diseases included. One of the many therapies that may be used is to reflow the obstruction of blood. Percutaneous coronary intervention (PCI) is one of them, and now it is claimed as the most advantageous reperfusion of the coronary arteries: Failure of this therapy may occur, with the no-reflow phenomenon being most common. Smoking may induce the no-reflow phenomenon and may lead to heart failure, increasing the risk of cardiogenic shock.

## Data availability

All data underlying the results are available as part of the article and no additional source data are required.

## Consent

Oral informed consent for publication of their clinical details and/or clinical images was obtained from the patient. Oral rather than written consent was obtained because of the patient’s condition and education level of the family, and was approved by the ethical review board of West Nusa Tenggara Province hospital.
