# Supplementary material for: Case Report: Smoking as the risk factor of persistent STEMI after primary percutaneous coronary intervention: how it could be happen?
Source: F1000Res. 2024 Oct 29;11:801. Originally published 2022 Jul 18. [Version 3] doi: 10.12688/f1000research.109757.3 (PMC11528186; doi:10.12688/f1000research.109757.3)
Supplement: Peer review history

# Reviewer report: Reviewer response for version 3

**DOI:** 10.5256/f1000research.173400.r336149

**Published:** 2024-10-31

**Authors:** İbrahim Rencüzoğulları

**Affiliations:** 1 Department of Cardiology, Kafkas University School of Medicine, Kars, Turkey

Dear editor

 The changes made and the answers given are satisfactory.

 My suggestion regarding the manuscript is accepted.

 Best regards.

Are enough details provided of any physical examination and diagnostic tests, treatment given and outcomes?

Yes

Is the case presented with sufficient detail to be useful for other practitioners?

Partly

Is sufficient discussion included of the importance of the findings and their relevance to future understanding of disease processes, diagnosis or treatment?

Yes

Is the background of the case’s history and progression described in sufficient detail?

Yes

Reviewer Expertise:

Cardiology, Hypertension, Coronary artery disease, Arrhythmia.

I confirm that I have read this submission and believe that I have an appropriate level of expertise to confirm that it is of an acceptable scientific standard.

# Reviewer report: Reviewer response for version 2

**DOI:** 10.5256/f1000research.152156.r199117

**Published:** 2023-10-03

**Authors:** İbrahim Rencüzoğulları

**Affiliations:** 1 Department of Cardiology, Kafkas University School of Medicine, Kars, Turkey

1. No-reflow phenomenon or short-term complications were not significantly different between current smokers and non smokers in the study of Shemirani H et al ( ***Ref:*** *Shemirani H, Tafti FD, Amirpour A. Comparison of no-reflow phenomenon after percutaneous coronary intervention for acute myocardial infarction between smokers and nonsmokers. J Res Med Sci. 2014 Nov;19(11):1068-73. PMID: 25657753; PMCID: PMC4310081*). In your study, however, it is not possible to confirm or falsify this result from a single case.
2. According to current guidelines, new generation and more potent P2Y receptor inhibitors such as ticagrelor and prasugrel should be given to this patient, but clopidogrel was given and the recommended 600 mg dose of clopidogrel was not given.
3. How accurate is it to give blood pressure medications such as bisoprolol (beta blocker), nitroglycerin and spironolactone to the patient in cardiogenic shock?
4. Anatomical nomenclature from angiography and echocardiography figures will be more beneficial and facilitating for the reader.
5. Spelling errors in the article should be corrected and spelling rules should be observed.
6. English of the article should be improved.

Are enough details provided of any physical examination and diagnostic tests, treatment given and outcomes?

Yes

Is the case presented with sufficient detail to be useful for other practitioners?

Partly

Is sufficient discussion included of the importance of the findings and their relevance to future understanding of disease processes, diagnosis or treatment?

Yes

Is the background of the case’s history and progression described in sufficient detail?

Yes

Reviewer Expertise:

Cardiology, Hypertension, Coronary artery disease, Arrhythmia.

I confirm that I have read this submission and believe that I have an appropriate level of expertise to confirm that it is of an acceptable scientific standard, however I have significant reservations, as outlined above.

## References

[1] : Comparison of no-reflow phenomenon after percutaneous coronary intervention for acute myocardial infarction between smokers and nonsmokers. *J Res Med Sci* .2014;19(11) :1068-73 25657753 PMC4310081

# Response

**Published:** 2024-10-12

**Authors:** Ricky Setiadi Yusuf

**Affiliations:** Mataram University, Indonesia

1. **No-reflow phenomenon or short-term complications were not significantly different between current smokers and non smokers in the study of Shemirani H et al ( *Ref: Shemirani* *H, Tafti FD, Amirpour A. Comparison of no-reflow phenomenon after percutaneous coronary intervention for acute myocardial infarction between smokers and nonsmokers. J Res Med Sci. 2014 Nov;19(11):1068-73. PMID: 25657753; PMCID: PMC4310081*). In your study, however, it is not possible to confirm or falsify this result from a single case.**

In this manuscript, we have already mentioned that the process by which the no-reflow phenomenon (NRP) occurs has not been described clearly. As in the present case, many factors can influence it, including smoking. In addition, leukocyte infiltration, vasoconstriction, activation of inflammatory pathways, and cellular edema are associated with the occurrence of NRP.

**2. According to current guidelines, new generation and more potent P2Y receptor inhibitors such as ticagrelor and prasugrel should be given to this patient, but clopidogrel was given and the recommended 600 mg dose of clopidogrel was not given.**

 Clopidogrel 300 mg was given in the emergency department, and was added 300 mg in the cathlab (total clopidogrel 600 mg)

**3. How accurate is it to give blood pressure medications such as bisoprolol (beta blocker), nitroglycerin and spironolactone to the patient in cardiogenic shock?**

 After the hemodynamic was stable, we started to give some medicine from low dose such as bisoprolol 2.5 mg/day, ramipril 2.5 mg/day, furosemide 20 mg/day, spironolactone 25 mg/day, atorvastatin 40 mg/day, aspirin 80 mg/day, nitroglycerin 10 mg/minute.

**4. Anatomical nomenclature from angiography and echocardiography figures will be more beneficial and facilitating for the reader.**

 In this revision, we have added the anatomical nomenclature.

**5. Spelling errors in the article should be corrected and spelling rules should be observed.**

 Thank you for your review, we already revised the spelling errors and the english

**6. English of the article should be improved.**

# Reviewer report: Reviewer response for version 1

**DOI:** 10.5256/f1000research.121300.r153430

**Published:** 2022-11-08

**Authors:** Mario Enrico Canonico

**Affiliations:** 1 Department of Advanced Biomedical Sciences, Federico II University, Naples, Italy

The Authors show the manuscript entitled "Case Report: Smoking as the risk factor of persistent STEMI after primary percutaneous coronary intervention: how it could be happen?"

 Some comments:

- How did the Authors assessed the no-reflow? Echocardiography is not useful for this purpose. Moreover, echocardiography can't make coronary artery disease diagnosis
- No-reflow depends by several factors (e.g. platelet reactivity, comorbidities)
- Why didn't the Authors perform a second coronary angiography? In order to assess acute complications as stent thrombosis.
- Images quality on EKG and Echo should be improved

Are enough details provided of any physical examination and diagnostic tests, treatment given and outcomes?

No

Is the case presented with sufficient detail to be useful for other practitioners?

No

Is sufficient discussion included of the importance of the findings and their relevance to future understanding of disease processes, diagnosis or treatment?

No

Is the background of the case’s history and progression described in sufficient detail?

No

Reviewer Expertise:

Clinical Cardiology

I confirm that I have read this submission and believe that I have an appropriate level of expertise to state that I do not consider it to be of an acceptable scientific standard, for reasons outlined above.

# Reviewer report: Reviewer response for version 1

**DOI:** 10.5256/f1000research.121300.r148891

**Published:** 2022-09-13

**Authors:** Cut Aryfa Andra

**Affiliations:** 1 H. Adam Malik Hospital, Medan, Indonesia; 2 Department of Cardiology and Vascular Medicine, Faculty of Medicine, Universitas Sumatera Utara, Medan, Indonesia

This case report is interesting and deserves to be studied, where it is said that many chemicals in cigarettes can cause lipid oxidation so that plaque deposits. The article explained that there can be a no-reflow phenomenon (NRP), where there is no return of coronary flow after percutaneous coronary intervention, in patients with smoking habits. Smoking can cause hypercoagulable states, decreased endothelial function, decreased oxygen supply, increased inflammation, platelet aggregation, thromboxane production, plasma thickening, and the amount of fibrinogen, thereby increasing the risk of plaque formation.

 The author adds insight to us that smoking is one of the important risk factors for heart attacks, both in the case of no backflow or no reflow phenomena. We need to realize that both in developing and developed countries, there are still many people who smoke and we should provide education about the dangers of smoking.

 In addition, some words may need to be revised.

1. In the introduction session, there was a typo in the lack of "Electrocradiogram"
2. In case report section, “…..He is a high school teacher with no same medical or familial, or psychosocial history before….” It appears that **before **may be unnecessary in this sentence, consider removing it.
3. In case report section , ‘’…Blood examination was measured, and there was no abnormality..’’ It seems that you should add **finding** after the word abnormality
4. In case report section , “..Coronary angiography was then done and confirmed that there was total occlusion in left anterior descending artery…” It appears that **then** may be unnecessary in this sentence, consider removing it.
5. In case report description, please add the picture of angiography result
6. In the case description “…After two days hospitalization, the patient developed to cardiogenic shock and lead to acute decompensated heart failure. It seem that you are missing preposition “the “. It’s considered to add the before hospitalization..”
7. In the case description “Two days after PCI, the patient complained of chest pain **again**. It seem that better to use recurrent chest pain”
8. In Discussion , last sentence”….This will reduce contractility function, increasing the risk of acute pulmonary edema and acute heart failure…” It seem that you are missing preposition “the “. It’s considered to add **the** before contractility.
9. In the discussion session, data from Shemirani et al., showed that the incidence of the no-reflow phenomenon was not significantly different between smokers and non-smokers, it could be revised by adding "s" to be non smokers.

Are enough details provided of any physical examination and diagnostic tests, treatment given and outcomes?

Partly

Is the case presented with sufficient detail to be useful for other practitioners?

Yes

Is sufficient discussion included of the importance of the findings and their relevance to future understanding of disease processes, diagnosis or treatment?

Yes

Is the background of the case’s history and progression described in sufficient detail?

Yes

Reviewer Expertise:

Cardiovascular intervention

I confirm that I have read this submission and believe that I have an appropriate level of expertise to confirm that it is of an acceptable scientific standard.
